# Diabetic Ketoacidosis Complicated With Extensive Rhinocerebral Mucormycosis and Bilateral Amaurosis in a Pediatric Patient

**DOI:** 10.7759/cureus.96582

**Published:** 2025-11-11

**Authors:** Nathália C Bertazzo, Guilherme G Filho, Larissa I Lunkes, Mauro A Czepielewski, Ticiana C Rodrigues

**Affiliations:** 1 Pediatrics, Hospital de Clínicas de Porto Alegre, Porto Alegre, BRA; 2 Pediatric Endocrinology, Hospital de Clínicas de Porto Alegre, Porto Alegre, BRA; 3 Endocrinology, Hospital de Clínicas de Porto Alegre, Porto Alegre, BRA

**Keywords:** antifungal treatment, diabetic ketoacidosis, mucormycosis, rhinocerebral mucormycosis, type i diabetes mellitus

## Abstract

Mucormycosis is a rare invasive fungal infection characterized by rapid progression and high mortality, caused by fungi of the order *Mucorales*. Patients with diabetes and inadequate glycemic control, particularly those in ketoacidosis, constitute an important risk group. We report the case of a 10-year-old boy admitted with diabetic ketoacidosis, who developed behavioral changes and bilateral visual loss. Imaging studies confirmed invasive fungal rhinosinusitis with intracranial extension and orbital and cerebral involvement. Treatment included surgical debridement and prolonged antifungal therapy (liposomal amphotericin B and isavuconazole). The patient was discharged from the hospital after 142 days of hospitalization, with irreversible visual loss. This case highlights the need for immediate recognition of orbital or neurological signs associated with rhinocerebral mucormycosis. Early clinical recognition, appropriate antifungal therapy, aggressive surgical debridement, and intensive glycemic control are fundamental pillars of treatment and influence the prognosis.

## Introduction

Mucormycosis is a rare invasive fungal infection caused by fungi of the order *Mucorales*, characterized by rapid progression and a high mortality rate. It predominantly affects immunocompromised individuals, such as patients with decompensated diabetes mellitus, hematologic malignancies, or those using immunosuppressants, and is uncommon in immunocompetent individuals [1-3]. Various organs and tissues can be involved, including the lungs, skin, and gastrointestinal tract, with the most frequent presentation being rhinocerebral involvement. There are few reports in the literature on mucormycosis with rhinocerebral involvement in children, and diagnosis can be especially difficult in them. Early diagnosis and initiation of antifungal therapy, in addition to surgical debridement, are essential to improve prognosis [3].

The objective of this article is to report the case of a child who was diagnosed with type 1 diabetes mellitus (DM1) and evolved with bilateral visual loss. The study was approved by Hospital de Clínicas de Porto Alegre Research Department (approval no. 2025/0373).

## Case presentation

A previously healthy 10-year-old male patient presented with a clinical picture characterized by polyuria, abdominal pain, and weight loss over three months, with a history of multiple medical visits. His condition progressed with worsening abdominal pain, intense frontal headache, purulent nasal discharge, and somnolence. He sought care at an urgent care unit, where he was admitted and diagnosed with severe diabetic ketoacidosis. Initial laboratory evaluation showed a pH of 6.95 (reference pH: 7.35-7.45), bicarbonate 4.3 mmol/L (reference: 22-26 mmol/L), and glycated hemoglobin (HbA1c) of 17% (reference: <5.7%). Treatment with insulin via continuous infusion was instituted, with resolution of ketoacidosis within 48 hours.

Despite correction of diabetic ketoacidosis, the patient developed neurological symptoms, characterized by behavioral changes, including drowsiness, agitation, and aggressiveness. A computed tomography (CT) scan of the brain revealed sinusitis without abnormalities in the central nervous system (CNS), and intravenous antibiotic therapy with vancomycin and cefepime was initiated. Approximately 48 hours later, the patient developed bilateral blindness and periorbital edema, with persistent altered mental status. Further investigation with magnetic resonance imaging (MRI) of the brain and orbits demonstrated findings suggestive of invasive fungal sinusitis with extension to the frontal lobes. Amphotericin B deoxycholate and metronidazole were added to the treatment regimen, and transfer to a higher-complexity hospital was requested.

**Figure 1:**
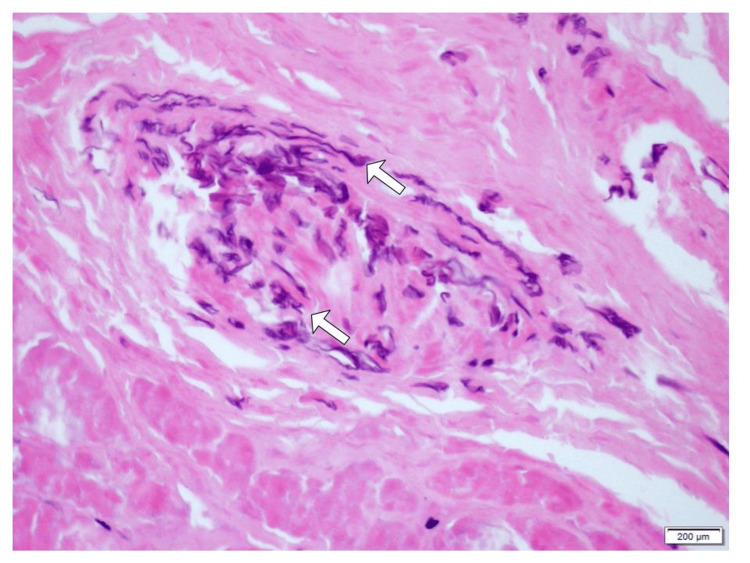
Histological section of the nasal septum, stained with hematoxylin and eosin (H&E), at intermediate magnification Wide, irregular, and non-septate hyphae are observed, consistent with a fungus of the order *Mucorales*, in addition to an area of ​​tissue necrosis.

The patient was transferred to the Hospital de Clínicas de Porto Alegre (HCPA) for further investigation and continuation of treatment. He was evaluated by the otorhinolaryngology team, who performed rigid endoscopic rhinoscopy and identified purulent secretion in both nasal cavities, as well as necrotic areas of the nasal septum. Given the high diagnostic suspicion of mucormycosis, the antifungal regimen was changed to liposomal amphotericin B, with a dose increase up to 10 mg/kg/day. The patient underwent sinusotomy and resection of the left nasal septum, middle turbinate, ethmoid colon, and lamina papyracea, in addition to the collection of material for anatomopathological (Figure 1) and microbiological analysis.

Culture results were positive for *Rhizopus oryzae*, confirming the diagnosis of rhinocerebral mucormycosis. During treatment, the patient developed a urticarial skin rash attributed to amphotericin, which was subsequently replaced by posaconazole. He progressed with irreversible visual loss. Ophthalmology evaluation revealed no evidence of intraocular fungal involvement, and a conservative approach was recommended due to the high morbidity associated with surgical intervention, as bilateral exenteration would have been required.

**Figure 2:**
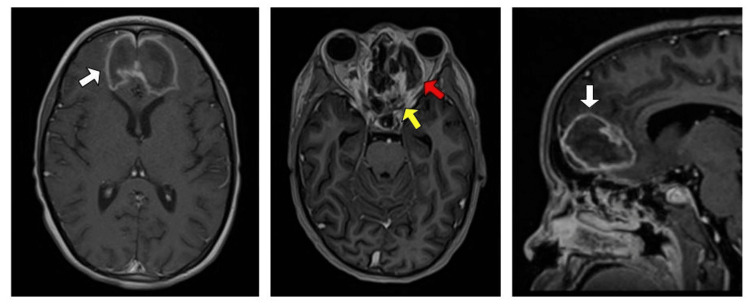
MRI of the skull showing signs of invasive fungal sinusitis with extension to the central nervous system and orbital cavities Note the mucosal thickening in the left maxillary and sphenoid sinuses (yellow arrow); extensive heterogeneous hypovascular area involving the frontal lobe, cribriform plate, and olfactory bulb, infiltrating the medial portions of the adjacent frontal lobes and anterior portion of the corpus callosum (white arrows); areas of necrosis within the orbital cavities, with infiltration of the ocular muscles and proptosis (red arrow).

Despite the instituted antifungal and surgical treatments, the patient developed recurrent episodes of severe headache requiring opioid analgesia. Consequently, a new brain MRI scan (Figure 2) was performed, revealing progression of the sinus disease, central nervous system involvement, and orbital cavity extension.

Given the clinical and radiological deterioration, liposomal amphotericin B therapy was resumed, and a neurosurgical craniotomy was performed for drainage of a bifrontal brain abscess and corticectomy. The collected material was sent for analysis, which identified hyaline hyphae in the frontal lobe specimen. In the postoperative period, the patient developed a bacterial infection and cerebrospinal fluid fistula as complications. Cultures grew *Enterobacter hormaechei*, requiring prolonged antibiotic therapy with cefepime, meropenem, and linezolid.

**Figure 3:**
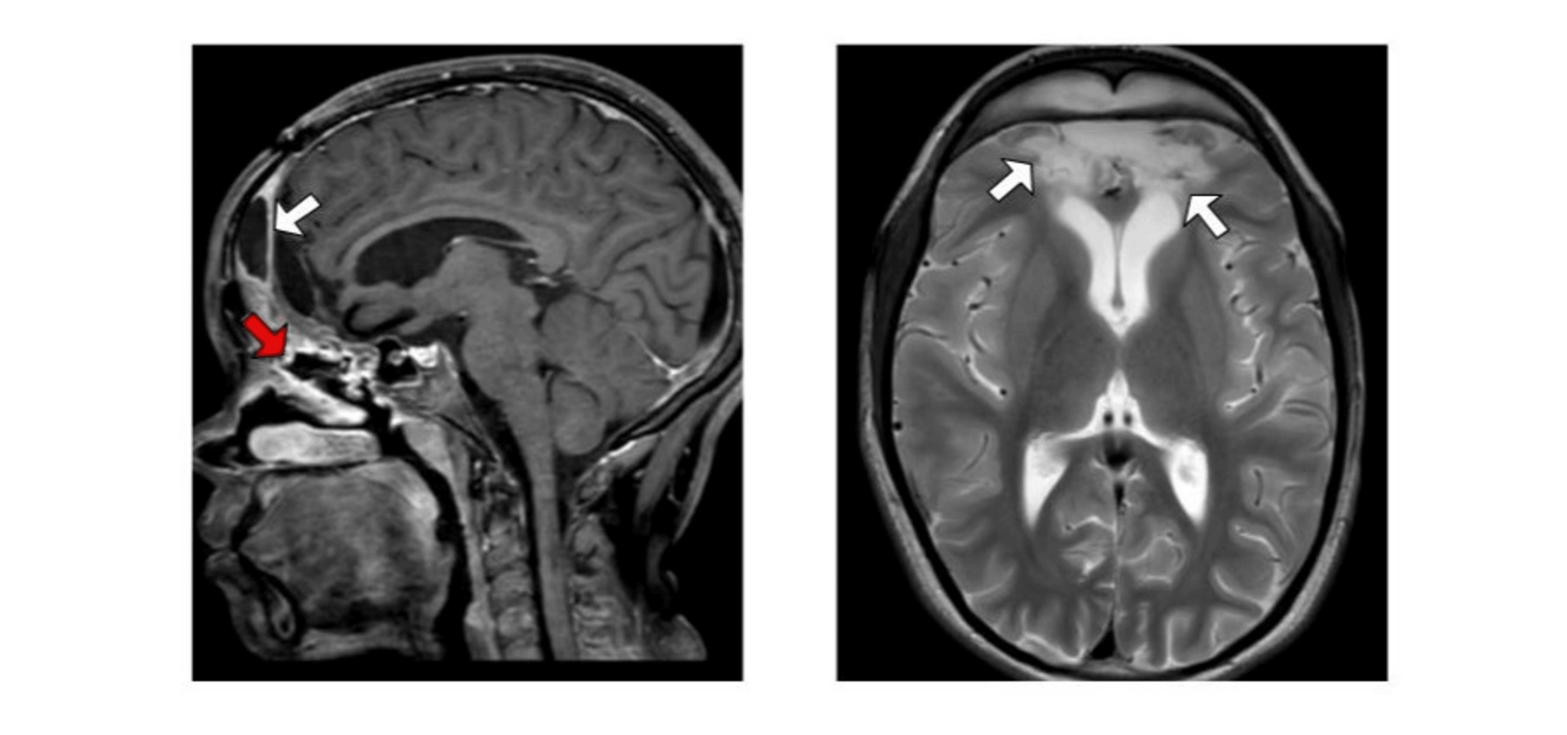
Magnetic resonance imaging of control skulls (four months after hospital discharge) Findings consistent with post-operative changes, following frontal craniotomy; encephalomalacia and cavity in the anterior aspect of the frontal lobes (white arrows); and mucosal thickening of the maxillary and frontal sinuses (red arrow).

During hospitalization, the patient received liposomal amphotericin B for 115 days, followed by transition to oral maintenance therapy with isavuconazole, with an initial loading dose of 200 mg every eight hours and a maintenance dose of 200 mg daily. Hospital discharge occurred after 142 days, with an indication to continue antifungal therapy at home for an indefinite period. Currently, 10 months after discharge, the patient is 12 years old, well adapted despite visual loss, on isavuconazole treatment, with no signs of progression of the fungal disease. Control MRI images are shown in Figure 3.

He is using insulin lispro and glargine and has good glycemic control, with the most recent HbA1c of 6.9%. He remains in multidisciplinary outpatient follow-up with pediatrics, pediatric endocrinology, infectious diseases, and neurosurgery teams.

## Discussion

Mucormycosis is a potentially lethal infection with a propensity for vascular involvement, thrombosis, and tissue necrosis. It is caused by filamentous fungi of the order *Mucorales*, formerly belonging to the class *Zygomycetes;* hence, the infection is also referred to as zygomycosis. The species most frequently isolated in clinical cases is* Rhizopus arrhizus *[3,4].

Regarding the risk factors for mucormycosis, the literature demonstrates a strong association with DM, particularly in the presence of diabetic ketoacidosis. A review of 929 reported cases of mucormycosis between 1940 and 2003 showed that DM was the most prevalent risk factor, present in 36% of cases, followed by hematologic malignancies (17%) and transplant recipients (12%) [5]. In a 2013 review of pediatric mucormycosis cases, DM1 was found in 53% of the infected patients [2]. There are also reports of mucormycosis presenting as the first manifestation of diabetes [4]. This predisposition, which is multifactorial in nature, is related to impaired neutrophil function caused by hyperglycemia, combined with the acidic environment of ketoacidosis that promotes fungal growth [1,6]. Rhizopus species, including the one isolated from the patient in this case, produce the enzyme ketone reductase, which favors their growth in acidic and hyperglycemic conditions [7]. Other predisposing factors include renal failure, prolonged use of corticosteroids, and immunosuppressive therapy [2,6].

Clinical manifestations depend on the site involved, which may be cutaneous, gastrointestinal, pulmonary, rhino-cerebral, disseminated, or uncommon forms such as endocarditis, osteomyelitis, peritonitis, and renal involvement. Rhinocerebral involvement is the most frequent presentation in children with diabetes [4,8]. This form of mucormycosis results from inhalation of environmental fungal spores or, less commonly, from loss of skin integrity, with rapid progression to adjacent tissues [3,4].

Cases of rhinocerebral mucormycosis initially present as sinusitis and periorbital cellulitis. In patients with risk factors, cranial nerve palsies, periorbital or facial pain, orbital inflammation, proptosis, headache, ophthalmoplegia, and acute loss of visual acuity are suggestive signs and symptoms for diagnosis. Fever may be absent in about half the patients [4]. Intracranial involvement arises from the orbital apex or the cribriform plate of the ethmoid bone, has devastating potential, and manifests as loss of consciousness, seizures, headache, hemiparesis, and gait disturbances [1,3,4].

Imaging assessment, such as CT and MRI, is essential to determine the extent of involvement in rhinocerebral mucormycosis. The main radiologic findings include mucosal thickening of the paranasal sinuses, nasal cavity, and paranasal regions, as well as destruction of periorbital tissues. Intracranial compromise may manifest as cerebral infarctions, fluid collections, cavernous sinus thrombosis, and involvement of the internal carotid artery, with possible thrombosis or occlusion [3,4].

Therapeutic management is based on two main pillars: antifungal therapy and surgical intervention. Liposomal amphotericin B is considered the first-line drug and is typically administered as monotherapy during the initial phase of treatment. The addition of other antifungal agents, such as isavuconazole or posaconazole, is recommended in cases of disease progression despite initial therapy. The duration of treatment is not strictly defined and varies according to several clinical factors, including the extent of lesions, patient age, response to antifungal therapy, and timing of surgical intervention [1,9,10]. 

Isavuconazole is a broad-spectrum triazole antifungal with documented efficacy against filamentous fungi, including species of the genera *Mucorales* and *Aspergillus*. It stands out for its oral formulation, which favors the continuity of treatment in an outpatient setting. The drug has good penetration into the CNS, making it a possibility for the treatment of rhinocerebral mucormycosis. Furthermore, it can be used as maintenance therapy after the initial use of parenteral antifungals, such as amphotericin B [10-12]. Another antifungal described in cases of mucormycosis is posaconazole, which has a broad spectrum against filamentous fungi. It also has an oral formulation and can be used as rescue therapy, with a success rate between 60-70% [6,9].

Cases of mucormycosis in pediatric patients with DM1 have been previously described in the literature. In the report by Masmoudi et al. [1], the patient presented with facial edema, rhinorrhea and diplopia in the context of diabetic ketoacidosis, and was treated with antifungal and surgery, with good clinical evolution. In another case, published by di Coste et al., in 2013 [6], a patient with DM1, diagnosed 11 years ago, with inadequate glycemic control, presented with dental pain, facial edema, and reduced visual acuity. She received surgical and drug treatment with posaconazole and progressed with visual loss.

The mortality rate is higher in patients with the cerebral, gastrointestinal, and disseminated forms [9). Prognosis is worse in cases with delayed initiation of antifungal therapy, intracranial extension, and visual loss at diagnosis [3].

## Conclusions

Early diagnosis and strict long-term glycemic control in DM are essential to prevent acute complications such as diabetic ketoacidosis, a major risk factor for severe opportunistic infections like mucormycosis. In patients with diabetes, especially in the presence of ketoacidosis, the manifestation of clinical signs such as sudden visual deterioration, orbital cellulitis, proptosis, ophthalmoplegia, or peripheral facial palsy should raise strong clinical suspicion of rhinocerebral mucormycosis and prompt immediate diagnostic investigation.

Rhinocerebral mucormycosis is a medical emergency with high morbidity and mortality. Early clinical recognition, together with appropriate antifungal therapy, aggressive surgical debridement, and intensive glycemic control, is the fundamental pillar of management. Continuous clinical surveillance and specialized outpatient follow-up are essential for rehabilitation and maintenance of quality of life, even in the face of irreversible sequelae such as visual loss. The reported case highlights that any child with diabetes who presents with sinusitis or orbital symptoms should be urgently evaluated for possible mucormycosis, in order to prevent irreversible complications.

## References

[1] Masmoudi M, Hasnaoui M, Ben Abdeljalil N (2021). Rhino-orbital cerebral mucormycosis in a child with type 1 diabetes: a case report. SAGE Open Med Case Rep.

[2] Berdai MA, Labib S, Harandou M (2016). Rhinocerebral mucormycosis complicating ketoacidosis diabetes (Article in French). Presse Med.

[3] Singh P, Arora S, Mittal N (2021). Diabetes and rhino-orbito-cerebral mucormycosis - a deadly duo. J Diabetes Metab Disord.

[4] Petrikkos G, Skiada A, Lortholary O, Roilides E, Walsh TJ, Kontoyiannis DP (2012). Epidemiology and clinical manifestations of mucormycosis. Clin Infect Dis.

[5] Roden MM, Zaoutis TE, Buchanan WL (2005). Epidemiology and outcome of zygomycosis: a review of 929 reported cases. Clin Infect Dis.

[6] di Coste A, Costantino F, Tarani L (2013). Rhinocerebral zygomycosis with pansinusitis in a 14-year-old girl with type 1 diabetes: a case report and review of the literature. Ital J Pediatr.

[7] GA GR, WE AM (1961). Studies of opportunistic fungi. I. inhibition of Rhizopus oryzae by human serum. Am J Med Sci.

[8] Moye J, Rosenbloom AL, Silverstein J (2002). Clinical predictors of mucormycosis in children with type 1 diabetes mellitus. J Pediatr Endocrinol Metab.

[9] Zaoutis TE, Roilides E, Chiou CC (2007). Zygomycosis in children: a systematic review and analysis of reported cases. Pediatr Infect Dis J.

[10] Brigmon MM, Ochoa B, Brust K (2021). Successful long-term therapy of mucormycosis with isavuconazole. Proc (Bayl Univ Med Cent).

[11] Gunathilaka SS, Keragala RK, Gunathilaka KM, Wickramage S, Bandara SR, Senevirathne IS, Jayaweera AS (2025). Use of isavuconazole in mucormycosis: a systematic review. BMC Infect Dis.

[12] Tsisar S, Salvado de Morais M, Gameiro R, Rodrigues M (2025). Successful management of rhino-orbital mucormycosis in a diabetic patient: a case report. Cureus.

